# Time-dependent improvement in functional outcome following Oxford medial unicompartmental knee arthroplasty: A prospective longitudinal multicenter study involving 96 patients

**DOI:** 10.3109/17453674.2011.652890

**Published:** 2012-02-08

**Authors:** Tor Kjetil Nerhus, Stig Heir, Ida Svege, Inge Skråmm, Tore Jervidalo, Jan Erik Madsen, Arne Ekeland

**Affiliations:** ^1^Orthopaedics Department, Martina Hansens Hospital, Baerum; ^2^Orthopaedics Department, Akershus University Hospital, Loerenskog; ^3^Orthopaedics Department, Lovisenberg Diaconal Hospital, Oslo; ^4^Orthopaedic Centre, Oslo University Hospital and Faculty of Medicine, University of Oslo, Oslo, Norway

## Abstract

**Background and purpose:**

10-year survival rates after unicompartmental knee replacement (UKR) have been up to 97% in single-center studies, but they have been as low as 80% in studies from arthroplasty registers. Few studies have evaluated short-term functional outcome and its improvement with time. We determined the time course of functional outcome as evaluated by the knee injury and osteoarthritis outcome score (KOOS) over the first 2 years after Oxford medial UKR.

**Patients and methods:**

In a prospective multicenter study, we included 99 unselected knees (96 patients, mean age 65 (51–80) years, 57 women) operated with Oxford medial UKR at 3 hospitals in the southeast of Norway between November 2003 and October 2006. Data were collected by independent investigators preoperatively and at 6 weeks, 3 months, 6 months, 1 year, and 2 years postoperatively. KOOS and range of motion (ROM) were determined at all follow-ups.

**Results:**

Mean KOOS values for pain and activities of daily living were improved already after 6 weeks, and increased between each time point up to 2 years postoperatively. However, no statistically significant improvements were seen after 6 months. Mean active and passive ROM gradually improved up to 2 years after UKR, and were then better than before surgery.

**Interpretation:**

Most of the expected improvements in pain and function after UKR are achieved within 6 months of surgery. Only minimal improvement can be expected beyond this time.

Unicompartmental knee replacement (UKR) has regained popularity in recent years. Studies comparing UKR to total knee replacement (TKR) have shown faster recovery, shorter hospital stay, more normal kinematics, better range of motion, less blood loss, fewer thromboembolic incidents, and fewer surgical site infections (Li et al. 2006, Walton et al. 2006, Furnes et al. 2007, Lombardi et al. 2009, Newman et al. 2009). Survival rates as high as 96–98% at 10 years have been reported in single-center studies (Murray et al. 1998, Pandit et al. 2011), whereas in studies from national arthroplasty registers 10-year survival rates have been reported to be as low as 80% (Furnes et al. 2007, Koskinen et al. 2008).

With a possible higher revision risk for UKR than for TKR (Furnes et al. 2007), more information on pain and function following knee arthroplasty is needed. During the last decade, several authors have emphasized the importance of measuring the patient's own experience of disability using self-reported questionnaires (Garratt et al. 2004, Tanner et al. 2007) such as the Oxford knee score, the knee injury and osteoarthritis outcome score (KOOS), and the International Knee Documentation Committee (IKDC) standard evaluation form.

We determined (1) the time course of patient-relevant functional outcome evaluated by the KOOS, and (2) the time course of range of motion (ROM) during the first 2 years following UKR using the Oxford medial unicompartmental knee prosthesis. Improvements in patients' self-reported pain and daily function during the study period were of particular interest.

## Patients and methods

96 unselected patients (99 knees) to be operated with medial unicompartmental knee arthroplasty at Martina Hansens Hospital, at Akershus University Hospital, and at Baerum Hospital between November 2003 and October 2006 were included in a prospective study. The inclusion criteria were: medial knee osteoarthritis, age 50–80 years, no rheumatoid arthritis or previous knee infection, a correctable varus deformity of less than 10°, a fixed flexion deformity of less than 10°, intact anterior and posterior cruciate ligaments, absence of osteoarthritis in the lateral compartment in standing radiographs, absence of lateral compartment tenderness, and no more than minimal patellofemoral symptoms or radiological abnormalities. Informed consent was obtained from all patients. All patients were operated with the Oxford phase III medial unicompartmental knee (Biomet Ltd., Bridgend, UK). The operation was performed in the hanging-leg position under spinal/epidural anesthesia, using a minimally invasive approach (Price et al. 2001). Mean operating time was 87 (52–152) min and 8 different surgeons were involved.

Data were collected by independent investigators (physiotherapists) preoperatively and at 6 weeks, 3 months, 6 months, 1 year, and 2 years postoperatively. The primary outcome measures were the KOOS subscales for pain and for ADL. Knee pain and knee daily function are among the most important clinical considerations when deciding whether or not to have a knee replacement; thus, these variables were assumed to be the 2 most important. The secondary outcome measures were the KOOS subscales for symptoms, knee-related quality of life (QoL), and sport and recreational function (see below); the Oxford score; the UCLA activity scale; and active and passive ROM.

### Knee injury and osteoarthritis outcome score (KOOS)

Patients completed the KOOS (Roos et al. 1998)—the Norwegian version (later published by Lygre and Furnes 2007)—on their own at each follow-up. The KOOS is a 42-item self-administered questionnaire based on the WOMAC osteoarthritis index (Bellamy et al. 1988), which has proven to be valid for subjects with total knee replacement (Roos and Toksvig-Larsen 2003). The KOOS has 5 subscales: Pain (9 items), other symptoms (7 items), activities of daily living (ADL) (17 items), sports and recreational function (Sport/rec) (5 items), and knee-related quality of life (QoL) (4 items). A score from 0 to 100 is calculated for each subscale, with 100 representing the best result. Patients were instructed to complete the KOOS form by considering their operated knee.

### Oxford knee score

Patients completed the Oxford knee score (Dawson et al. 1998) (Norwegian version) on their own at each follow-up. The Oxford knee score is a 12-item validated self-report questionnaire designed for patients who undergo knee replacement. The score assesses patient function and activities of daily living, scoring each activity from 0 to 4. A cumulative score from 0 to 48 is then calculated, with 48 representing the best result.

### UCLA activity scale

Patients also completed the UCLA activity scale (Zahiri et al. 1998) (Norwegian version) on their own at each follow-up. The UCLA activity scale is a simple self-report scale ranging from 1 to 10. Patients indicate their most appropriate activity level, with 1 defined as “no physical activity, dependent on others” and 10 defined as “regular participation in impact sports”.

### Range of motion (ROM)

Non-weight-bearing active and passive ROM values were obtained by an independent investigator (physiotherapist) with the patient in supine position in order to allow free hip flexion. A goniometer was used.

### Complications

Patients were asked to report any complications or adverse events at all follow-ups.

### Statistics

The minimal perceptible clinical improvement (MPCI) of the KOOS is suggested to be approximately 10 score units (Roos and Lohmander 2003). Previous data from a similar patient group (Nerhus et al. 2010) indicated that the mean difference in KOOS scoring between time points is normally distributed with a standard deviation of approximately 20. We found that 33 patients would be needed to obtain a power of 0.80 and a significance level of p < 0.05.

The data were analyzed by fitting separate linear mixed models for the KOOS subscales, for the Oxford score, for the UCLA score, and for the ROM measurements, choosing an unstructured (i.e. completely general) covariance matrix for the repeated measurements. Missing data were assumed to be missing at random. The mean of each outcome was estimated along with its corresponding 95% confidence interval (CI) at all observation times. Post hoc comparisons between the main effects of all pairs of points in time (that is, no reference category defined) were performed separately for each model, corresponding to the KOOS subscales, the Oxford score, the UCLA activity scale, and the measurements of active and passive ROM. Bonferroni adjustments, including all pairwise comparisons within a specific model, were applied to the respective confidence intervals and p-values to account for multiple testing. The level of statistical significance was set to p < 0.05. Statistical analysis was performed using SPSS software version 18.

## Results

Data from all 96 patients (99 knees) initially included in the study were available for analysis.

### Missing data

Of 594 evaluations planned, 38 (6%) were missing for 27 patients. 21 patients failed to attend 1 or more of the postoperative evaluations, 5 patients were revised to have a total knee replacement, and 1 patient died of gastrointestinal cancer shortly after the operation. The mean age, sex distribution, and KOOS values of these 27 patients were similar to those of the 69 patients with complete data.

### Complications

5 knees (5 patients) were revised to a total knee replacement during the study period: 2 due to pain, 2 because of aseptic loosening, and 1 because of a deep infection. 1 patient developed a stiff knee because of arthrofibrosis, and was treated with manipulation under anesthesia with a satisfactory result (ROM was 110 degrees at 2 years). 1 patient had a meniscal bearing dislocation caused by a hyperflexion trauma, and was reoperated with exchange of the meniscal bearing.

### KOOS

The linear mixed-models analysis revealed significant differences in KOOS scoring between the 6 time points of measurement. This was true for all subscales of the KOOS (p < 0.001).

**Figure 1.**
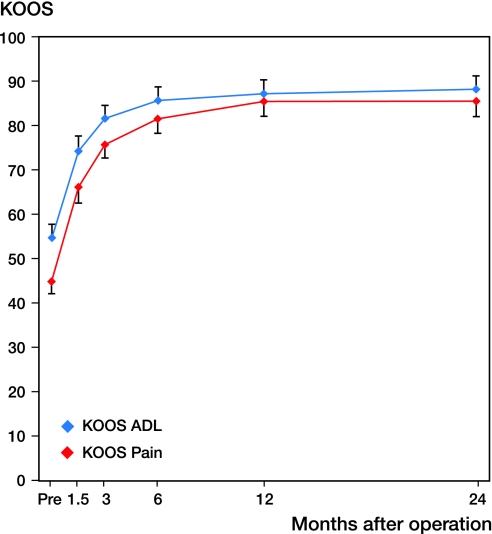
Improvement with time of KOOS subscales ADL and Pain. Pre = preoperatively. Values are mean and bars represent 95% CI. Pairwise comparisons revealed statistically significant improvement between Pre and all other time points for both subscales (p < 0.001 in both cases), between 6 weeks and 3 months (p < 0.001 in both cases), between 3 months and 6 months (p = 0.03 and p = 0.006), between 3 months and 1 year (p = 0.006 and p < 0.001), and between 3 months and 2 years (p < 0.001 in both cases).

Mean KOOS score values for ADL and for pain continued to improve until 1 year after surgery. Minimal changes were seen after 6 months (Figure 1). Using the 2-year scoring values as a reference, the percentage of improvement in KOOS pain achieved was 52% at 6 weeks, 76% at 3 months, 90% at 6 months, and 100% at 1 year. Likewise, the percentage of improvement in KOOS ADL achieved was 58% at 6 weeks, 81% at 3 months, 93% at 6 months, and 97% at 1 year.

**Figure 2.**
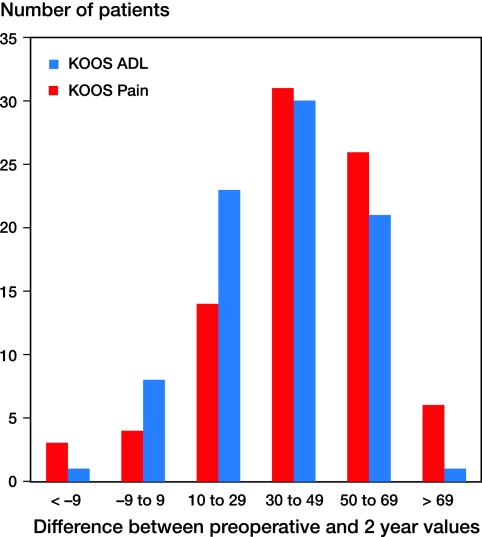
Patients with complete data at 2 years (n = 84) were divided into groups according to the magnitude of the difference between mean preoperative values and mean 2-year values in the KOOS subscales Pain and ADL. Differences of +10 or more score points are better than the suggested minimal perceptible clinical improvement (MPCI) (Roos and Toksvig-Larsen 2003).

Patients with complete KOOS data at 2 years (n = 84) were divided into groups according to the magnitude of the difference between mean preoperative values and mean 2-year values in the KOOS subscales pain and ADL (Figure 2). 75 patients (86%) achieved improvement of 10 or more KOOS ADL score points, whereas 77 patients (92%) achieved improvement of 10 or more KOOS pain score points.

**Figure 3.**
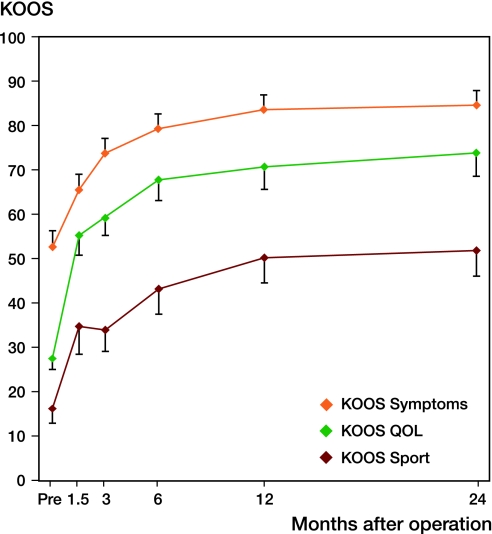
Improvement in KOOS subscales QoL, Symptoms, and Sport/rec with time. Values are mean and bars represent 95% CI. Pairwise comparisons revealed statistically significant improvement between Pre and all other time points (p < 0.001). This was true for all 3 subscales.

Mean KOOS score values for Symptoms, QoL, and Sport/rec also continued to improve until 2 years after surgery. Only small changes were observed after 6 months, however (Figure 3).

### Oxford score

**Figure 4.**
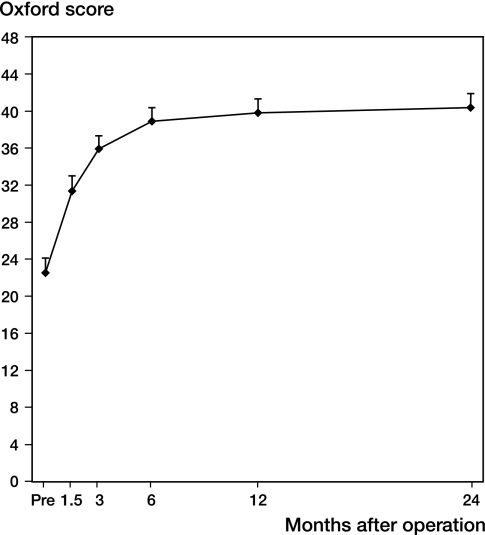
Improvement in Oxford score 0–48 with time. Values are mean and bars represent 95% CI. Pairwise comparisons revealed statistically significant improvement between Pre and all other time points (p < 0.001), between 6 weeks and 3 months (p < 0.001), between 3 months and 6 months (p=0.001), between 3 months and 1 year (p < 0.001), and between 3 months and 2 years (p < 0.001).

The linear mixed-models analysis revealed significant differences in Oxford scoring between the 6 times of measurement (p < 0.001). Oxford mean score values continued to improve until 2 years after surgery, but only small changes were seen after 6 months (Figure 4).

### UCLA activity scale

**Figure 5.**
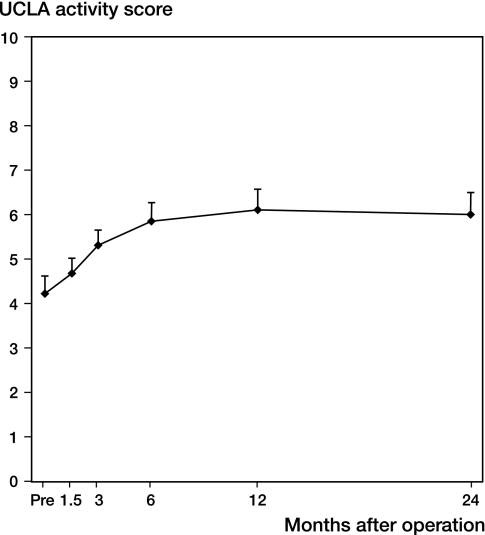
Improvement in UCLA activity score with time. Values are mean and bars represent 95% CI. Pairwise comparisons revealed statistically significant improvement between Pre and all other time points (p < 0.001), except 6 weeks (p = 0.724). There was also statistically significant improvement between 6 weeks and 3 months (p = 0.02), between 6 weeks and 6 months (p < 0.001), and between 3 months and 1 year (p = 0.03).

The linear mixed-models analysis revealed that there were significant differences in UCLA scoring between the 6 times of measurement (p < 0.001). UCLA mean score values continued to improve until 1 year after surgery, with only minimal changes observed after 6 months (Figure 5).

### Range of motion

**Figure 6.**
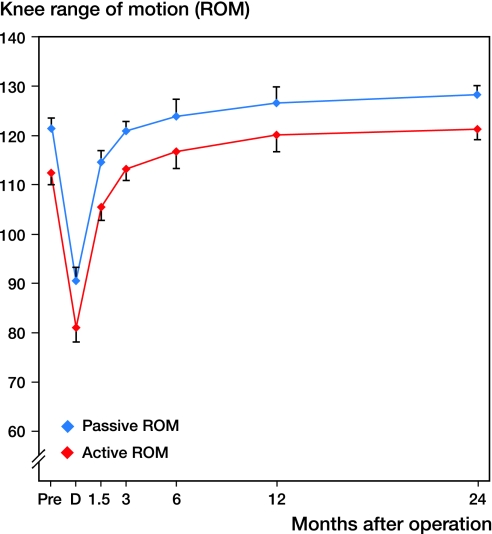
Improvement in active ROM (red line) and passive ROM (blue line) with time. D = discharge from hospital. Values are mean and bars represent 95% CI. Pairwise comparisons for active ROM revealed statistically significant differences between D and all other time points (p < 0.001), between 6 weeks and 3 months (p < 0.001), between 3 months and 1 year (p = 0.006), and between 6 months and 2 years (p = 0.04). For passive ROM, there were significant differences between D and all other time points (p < 0.001), between 6 weeks and 3 months (p < 0.001), between 3 months and 1 year (p = 0.04), and between 3 months and 2 years (p < 0.001).

The linear mixed-models analysis revealed significant differences in both active and passive ROM between the 7 times of measurement (p < 0.001). Mean values for active and passive ROM continued to improve until 2 years after surgery (Figure 6).

Patients were divided into groups according to their preoperative passive ROM, and mean preoperative ROM was compared to mean 2-year ROM for each group. Patients with a preoperative ROM of 80–99 degrees (n = 6) improved in mean ROM (from 95 to 122 degrees; p = 0.004), patients with a preoperative ROM of 100–119 degrees (n = 26) improved in mean ROM (from 112 to 122 degrees; p = 0.001), patients with a preoperative ROM of 120–129 degrees (n = 43) improved in mean ROM (from 124 to 130 degrees; p < 0.001), and patients with a ROM of 130–149 degrees preoperatively (n = 24) ended up with approximately the same mean ROM (from 133 to 134 degrees; p = 0.7) 2 years postoperatively.

## Discussion

To our knowledge, only 2 studies have used the KOOS to evaluate functional recovery after UKR (Lygre et al. 2010, Felts et al. 2010), and our study is the first to evaluate functional results after UKR using repetitive measures of the KOOS.

We found improvement in pain and function as early as 6 weeks postoperatively; more than 50% of the improvement in KOOS Pain and KOOS ADL had already been achieved at this point. Improvement in KOOS Pain and KOOS ADL mean values continued until 2 years postoperatively, although the differences between the 6-month and 2-year values were not statistically significant. The minimum perceptible clinical improvement (MPCI) in the KOOS is suggested to be approximately 10 score units (Roos and Lohmander 2003). The mean KOOS Pain score rose 4 points from 6 months to 2 years whereas the mean KOOS ADL score rose 2 points in the same time period. Considering this, the improvements in these KOOS subscales between 6 months and 2 years in our study are probably not clinically relevant. In terms of patient information, it would be fair to say that most of the improvement in pain and ADL after UKR is achieved at 6 months; further improvement can be expected to be minimal. Furthermore, between 92% (KOOS Pain) and 86% (KOOS ADL) of the patients experienced a clinically relevant improvement (10 points or more) from preoperatively to 2 years postoperatively (Figure 2). This suggests that about 10% of the patients either experienced no change or an aggravation after the operation. In their study on UKR, Pandit et al. (2011) found that 4% of the patients were not very pleased, and 2% were very disappointed. Nilsdotter et al. (2009) used the KOOS to study improvement after TKR, and found that 12% of the patients experienced a change in KOOS Pain of less than 10 points. This coincides with our findings on UKR. Regarding clinical function, a number of patients with UKR and TKR experience unsatisfactory results. This should be included in the preoperative patient information.

Paradowski et al. (2006) collected population-based reference data for the KOOS in different age groups. The reference data for KOOS ADL in the age group 55–74 years were 86 for men and 77 for women. For KOOS Pain in the age group 55–74 years, the reference data were 88 for men and 78 for women. Our results for KOOS ADL were actually somewhat better than the reference data whereas our results for KOOS Pain were similar. Lygre et al. (2010) recorded a KOOS Pain score of 77 and a KOOS ADL score of 75 at a minimum of 2 years after UKR. Our UKR results were somewhat better than these, with a KOOS Pain score of 86 and KOOS ADL score of 88 at 2 years. We cannot find any obvious reason for this difference. Contributing factors may have been that the mean age of their study population was slightly higher (69 years as opposed to 65 years), and that the patients were recruited from 42 hospitals, and it is possible that some had wider indications for the primary UKR.

Studies on total knee replacement indicate that the most important factor determining postoperative ROM is the preoperative ROM (Ritter et al. 2003, Gandhi et al. 2006, Nerhus et al. 2010). We found statistically significant improvement between mean preoperative ROM (both active and passive) and mean 2-year postoperative ROM. Furthermore, when the patients were divided into subgroups according to their preoperative ROM, the results showed that the stiffer the knees were preoperatively, the more ROM they gained compared to the 2-year results. Only those patients with the best preoperative ROM (130–149 degrees) remained unchanged compared to 2 years postoperatively.

During the last decade, several authors have emphasized the importance of measuring the patient's own experience of disability using self-reported questionnaires (Garratt et al. 2004, Tanner et al. 2007). Different scales have been developed that measure specific health considerations such as function during daily activities and pain. In a study to determine whether different knee-specific outcome instruments included items to detect symptoms and disabilities most important to the patients, the KOOS (Roos et al. 1998) and the International Knee Documentation Committee (IKDC) Standard Evaluation Form were identified as the top 2 general knee quality-of-life instruments ensuring that the patient's point of view was considered (Tanner et al. 2007). We used the Norwegian version of the KOOS, which was published by Lygre and coworkers (2007). To our knowledge, only a few studies on functional results after UKR have involved follow-up at multiple time points (Kleijn et al. 2007, Newman et al. 2009), and none of them have used self-reported questionnaires. One of the strengths of our study is that we have included both short- and medium-term evaluations, which are necessary to establish a true timeline of functional recovery.

The present study has some limitations. One weakness is that we had some missing data. However, when designing the study, we decided to use linear mixed-models statistics. This method makes allowances for some missing data, and all the patients who had been included could still be kept in the study. The analysis of missing data revealed only small differences between patients with missing data and patients with complete data sets. Another objection might be the method of measuring ROM—clinically with a goniometer. Ryd et al. (1997) found that clinical measurements of, for example, flexion and extension are of questionable reliability. Lenssen et al. (2007) showed that reliability of clinical ROM measurement was acceptable regarding group comparisons, but poor with regard to individual measurements over time. Measurements on radiographs may be a more reliable way to examine ROM (Lavernia et al. 2008), but they are more expensive and time consuming.

A study of functional results after UKR should preferably include both a patient-relevant self-evaluation score and more demanding objective tests. Kleijn et al. (2007) studied functional results at multiple time points after UKR using the clinician-based KSS score and the more objective Dynaport test. They found that the KSS score showed no statistically significant improvement after 6 months, whereas the Dynaport knee test showed improvement up to 2 years in one subscore, indicating that the more objective Dynaport knee test is more discriminative than the KSS score. ROM was the only objective parameter we studied.

In summary, we found that functional results and recovery after UKR are time-dependent. There was improvement in pain and function as early as 6 weeks postoperatively, and most of the expected improvements were achieved at 6 months. ROM gradually improved up to 2 years after UKR, and was then better than before surgery.
